# Multicentric Surveillance of Antimicrobial Resistance to Generate Data-Driven Regional Antibiograms: A Laboratory-Based Cross-Sectional Study in Pakistan

**DOI:** 10.3390/antibiotics14111154

**Published:** 2025-11-14

**Authors:** Nadia Noreen, Adeel Aslam, Mateen Abbas, Asma Ghulam Mustafa, Shazia Jamshed, Márió Gajdács, Ayesha Iqbal, Wajid Syed, Adel Bashatah, Naji Alqahtani

**Affiliations:** 1 Department of Pharmacy, University of Lahore, Lahore 54792, Pakistan; nadianoureen1980@gmail.com (N.N.); asmaghulammustafa281@gmail.com (A.G.M.); 2 Faculty of Pharmacy and Biomedical Sciences, Mahsa University, Jenjarom 42610, Malaysia; 3 Faculty of Pharmacy, Capital University of Science and Technology (CUST), Islamabad 44000, Pakistan; mateen.abbas@cust.edu.pk; 4 Department of Pharmacy Practice, School of Pharmacy, IMU (International Medical University), Kuala Lumpur 57000, Malaysia; shaziajamshed@imu.edu.my; 5 Department of Public Health, Albert Szent-Györgyi Faculty of Medicine, University of Szeged, 6720 Szeged, Hungary; gajdacs.mario@szte.hu; 6 Department of Pharmacy Practice and Policy, University Park Campus, University of Nottingham, Nottingham NG72RD, UK; aiqbal6@ualberta.ca; 7 Office of Lifelong Learning and the Physician Learning Program, Faculty of Medicine and Dentistry, University of Alberta, Edmonton, AB T6G1C9, Canada; 8 Department of Clinical Pharmacy, College of Pharmacy, King Saud University, Riyadh 11451, Saudi Arabia; 9 Department of Nursing Administration and Education, College of Nursing, King Saud University, Riyadh 11451, Saudi Arabia; abashatah@ksu.edu.sa (A.B.); alqnaji@ksu.edu.sa (N.A.)

**Keywords:** antibiotics, antimicrobial resistance, data-driven, antibiogram, surveillance, Pakistan

## Abstract

**Background**: The escalating burden of antimicrobial resistance (AMR) poses a critical threat to public health in Pakistan, with rates of high antibiotic consumption and limited standardized surveillance on AMR rates. Our study aimed to carry out a multicentric surveillance of AMR to generate regional antibiograms for Northern Punjab, Pakistan, to guide empirical antimicrobial therapy and stewardship efforts. **Methods**: A laboratory-based, retrospective cross-sectional study was conducted over a six-month period across three tertiary care hospitals. Socio-demographic, clinical, and microbiological data (including specimen type and antibiotic prescription rates) were collected from *N* = 485 patients with confirmed bacterial infections. Antimicrobial susceptibility testing was performed based on Clinical Laboratory Standards Institute (CLSI) recommendations. Statistical analyses were carried out using SPSS v.22.0. **Results**: In our study setting, Gram-positive bacteria were common causes (60.0%) of infections, with *Staphylococcus aureus* (12.2%) and *Streptococcus pneumoniae* (10.3%) being the most relevant. Among Gram-negative bacteria (40.0%), *Escherichia coli* (14.0%) and *Pseudomonas aeruginosa* (5.8%) were shown to be important pathogens. Overall, 25.0% of *S. aureus* isolates were methicillin-resistant (MRSA), while ~30% of *E. coli* showed resistance to third-generation cephalosporins (3GCs). Enterobacterales species had highly variable susceptibility rates (40–70%) for fluoroquinolones. Meropenem and vancomycin/linezolid retained high efficacy (>90%) against most Gram-negative and Gram-positive isolates, respectively. In all healthcare settings studied, ceftriaxone was the most frequently prescribed antibiotic. **Conclusions**: High levels of resistance against first-line antibiotics were noted in our setting of Northern Punjab, Pakistan, underscoring the critical need for robust antimicrobial stewardship programs, tailored to local institutional contexts, capabilities, and needs. The regional antibiogram developed based on our data may provide vital evidence for informing local empirical treatment guidelines, which need to be continuously updated.

## 1. Introduction

Since their introduction into routine clinical practice in the late 1940s, antibiotics (ABs) have become the foundation of contemporary medicine, including our capacity to ensure the successful management of previously life-threatening infections and enabling the advancement of numerous intricate medical disciplines and specialties [1,2]. These developments—including carrying out invasive surgical interventions, intensive care medicine, organ transplantations, chemotherapy for malignant disorders, and the care of preterm infants—has been threatened due to the emergence of antimicrobial resistance (AMR), reducing the available therapeutic arsenal of antimicrobial drugs to critical levels [3,4]. Due to the substantial burden of infections caused by multidrug-resistant (MDR) bacteria, many international organizations have called for global action to tackle this critical public health issue [5,6,7]. Based on the recent Global Burden of Disease (GBD) estimates, bacterial infections resistant to commonly used ABs are responsible for nearly 4.71 million deaths, with 1.14 million deaths directly attributed to AMR in 2021; furthermore, the report highlighted that by 2050, Africa and Asia will bear the majority of the AMR-related burden [8]. Low- and middle-income countries (LMICs), including Pakistan (classified as a lower MIC, according to the World Bank), bear a disproportionate burden due to a complex interplay of factors, including demographic characteristics, inadequate healthcare infrastructure, a high burden of infectious diseases, limited availability and adherence to infection prevention and control (IPC) measures, and the overuse and misuse of ABs, partly fueled by the widespread availability of non-prescription antibiotics [9,10]. Pakistan ranks among the highest AB-consuming countries globally, with an estimated 53% of prescriptions associated with ABs, many of which are used inappropriately [11,12]. As a result, according to the results of the Global Research on Antimicrobial Resistance [13] Project, almost 60,000 deaths occurred directly due to and an additional ~221,000 death were associated with bacterial AMR in 2019 [14]. Given the relevance and dangers of AMR, in 2024, the United Nations (UN) has adopted a political declaration against AMR, committing to reduce mortality associated with bacterial AMR by 10% by 2030 [15]. Similarly, the World Health Organization updated their 2017 list of Bacterial Priority Pathogens List (BPPL) [16] in 2024 [17], to guide global research and development efforts towards novel ABs. In addition, based on the recent decision of the World Health Assembly, the WHO Global action plan on AMR is scheduled to be updated in 2026 [18].

In any given country, the effective management of the consequences of AMR requires strong commitment from a policy perspective, the dedication of healthcare professionals, and the existence of relevant healthcare infrastructure [19,20]. The success of antimicrobial stewardship (AMS) programs—i.e., healthcare system-wide efforts dedicated to optimizing the use of available antimicrobial drugs—is majorly dependent upon the availability of reliable susceptibility data, resulting from standardized surveillance initiatives [21]. Surveillance of antimicrobial susceptibility patterns is critical for informing national treatment policies and optimizing empirical antibiotic selection [19,22]. While Pakistan participates in the WHO’s Global Antimicrobial Resistance and Use Surveillance System (GLASS), the lack of standardized AMR surveillance across numerous regions of Pakistan has made it difficult for clinicians to establish effective empirical treatment guidelines, leading to the persistence of habitual prescribing behaviors, an increased risk of treatment failure, prolonged hospital stays, and higher mortality rates [22]. Several studies have reported alarmingly high resistance rates among common Gram-negative bacteria, including *Escherichia coli* and *Klebsiella pneumoniae*, which exhibit resistance rates >70% for third-generation cephalosporins (3GCs) and 50% for carbapenems in some regions [23]. This tendency was also demonstrated among community-acquired Gram-negative infections, showing resistance rates of >70% fluoroquinolones, trimethoprim–sulfamethoxazole, and 3GCs [24]. Similarly, high rates (40–60%) of methicillin resistance (MR) were noted among *Staphylococcus aureus* isolates—both among hospital-acquired and community-associated infections—limiting the safe and effective treatment options for common Gram-positive infections [25]. Nonetheless, most existing AMR data in Pakistan are derived from single-center cross-sectional studies, which may not account for regional variations in AMR rates. Conversely, a multicenter approach would ensure a diverse and representative sample, allowing findings to be generalized across various geographical locations. Data-driven regional antibiograms, which aggregate antimicrobial susceptibility data from multiple healthcare centers with diverse profiles and patient populations, may provide evidence-based information to act as a decision aid, guiding empirical therapy, and as an actionable framework for IPC/AMS programs [26,27]. Given Pakistan’s diverse geographical and socio-economic landscape, multicentric AMR surveillance is essential to identify high-risk areas and trends in bacterial resistance [28,29]. Consequently, the present study aimed to conduct multicentric surveillance of AMR rates across diverse healthcare facilities in Northern Punjab, Pakistan—including antimicrobial prescription practices corresponding to patients affected by bacterial infections—to generate regional, data-driven antibiograms that may enhance clinical decision-making and aid AMS initiatives in the local context.

## 2. Results

### 2.1. Socio-Demographic Characteristics and Antimicrobial Prescriptions in the Study Population

Out of the *N* = 600 patients presenting with bacterial infections from the three healthcare institutions (Hospitals 1–3) during the study period, the microbiological data corresponding to (*N* = 485; 100.0%) were eligible for analysis; a summary of socio-demographic characteristics corresponding to these patients is presented in Table 1. The majority of affected patients were male (58.8%; *n* = 285), within the age bracket of 40-to-60 years (35.1%; *n* = 170). In total, 48.5% (*n* = 235) of patients were treated at the University of Lahore Teaching Hospital (Hospital 3), while the other share of patients was treated at public institutions (Hospital 1 and Hospital 2); 63.9% (*n* = 310) were treated as inpatients. The data collected during this study covered microbiological and patient data from multiple hospital departments, with patients from the internal medicine (12.4%; *n* = 60), gynecological (11.3%; *n* = 55), surgical, pediatric, and cardiological departments (10.3%; *n* = 50 each) being the most numerous (Table 1).

The distribution of specimens from the samples collected from the patients was as follows, reflecting their importance in diagnosing infections in clinical practice: the majority were collected from blood cultures (31.1%; *n* = 151), followed by urine specimens (24.9%; *n* = 121), sputum samples (16.6%; *n* = 80), wound swabs (10.3%; *n* = 50), pus (6.3%; *n* = 30), ear swabs (4.0%; *n* = 19), cerebrospinal fluid (2.8%; *n* = 14), pyogenic cultures (2.0%; *n* = 10), nasal swabs (1.1%; *n* = 6), and vaginal samples (0.9%; *n* = 4).

### 2.2. Distribution of Bacterial Isolates and Consumption of ABs

The distribution of bacterial isolates from clinically relevant positive cultures revealed a predominance of Gram-positive bacteria (60.00%; *n* = 291), with *S. aureus* being the most common (12.16%; *n* = 59), followed by other relevant pathogens, such as *Streptococcus pneumoniae* (10.31%; *n* = 50), *S. pyogenes* (7.22%; *n* = 35), *Enterococcus faecalis* (5.77%; *n* = 28), *Bacillus subtilis* (5.57%; *n* = 27), *B. cereus* (4.95%; *n* = 24), *Clostridium perfringens* (4.54%; *n* = 22), and *Listeria monocytogenes* (4.12%; *n* = 20) (Table 2). On the other hand, Gram-negative bacteria accounted for 40.00% (*n* = 194) of isolates, with *E. coli* being the most frequently detected (14.02%; *n* = 68), followed by *Pseudomonas aeruginosa* (5.77%; *n* = 28), *K. pneumoniae* (3.71%; *n* = 18), *Salmonella Enterica* (3.51%; *n* = 17), *Haemophilus influenzae* (2.68%; *n* = 13), *Neisseria gonorrhoeae* (1.65%; *n* = 8), and *N. meningitidis* (1.65%; *n* = 8), among others (Table 2). The distribution of bacterial isolates from clinically relevant infections stratified according to specimen type is shown in Figure S1.

Our study assessed and ranked the frequency and characteristics of AB prescriptions received by patients in Hospitals 1–3, and have highlighted the most commonly used drugs, as seen in Table 3. Overall, the 3GC ceftriaxone (CRO)—with a broad spectrum of activity—was the most commonly prescribed AB for patients treated in all departments, irrespective of age group or the type of hospital (Table 3). In addition, fluoroquinolone ABs (i.e., ciprofloxacin [CIP], moxifloxacin [MOX]) and metronidazole (MET) were among the ABs commonly prescribed during the study period. On the other hand, ABs with a narrow spectrum of activity were seldom seen among the five most commonly prescribed drugs. As seen in Table 3., the overwhelming majority of prescribed ABs belonged to the WHO’s “Watch” antibiotic group, with a considerable rate of use of “Reserve” drugs as well.

### 2.3. Antimicrobial Susceptibility Analysis of Clinically Relevant Bacteria

Table 4 and Table 5 summarize the antibiogram data, i.e., the ratio of susceptible (S%) isolates among clinically relevant Gram-positive and Gram-negative bacteria detected in the study, respectively. The colors in the table denote the number of isolates detected throughout the study period, i.e., the reliability and relevance of the findings in our local context. *S. aureus* largely retained its susceptibility to vancomycin (VAN) and linezolid (LZD) (S: 95.0%), while 25.0% of isolates were MRSA, i.e., resistant to most beta-lactam antibiotics. Similarly, in the cases of *S. pneumoniae* and *S. pyogenes*—in addition to most beta-lactam antibiotics—VAN and LZD showed high sensitivity rates. Conversely, in the case of *E. faecalis*, a more nuanced susceptibility situation is seen, with higher rates of resistant isolates for numerous tested antimicrobials (Table 4). In the context of Gram-negative bacteria, highly variable patterns of susceptibility across pathogens were seen, reflecting reliable treatment options. *E. coli* showed high sensitivity to piperacillin/tazobactam (PIP/TAZO; 90.0%) and meropenem (MER; 90.0%); overall, ~30% of isolates were resistant to 3GCs. For *K. pneumoniae*, a higher rate of resistance was seen for 3GCs and fluoroquinolones, but MER retained its effectiveness in the majority (90.0%) of isolates (Table 5). As a general rule, members of the Enterobacterales order had highly variable susceptibility to fluoroquinolones, with susceptibility ranges of 40–70%. In non-fermenting Gram-negative bacteria, such as *P. aeruginosa* and *A. baummannii*, MER susceptibility remained high, while resistance towards AMK (50.0% and 60.0%) and fluoroquinolones were considerably high. Among pathogenic *Neisseria* species, beta-lactams, such as CRO and cefoperazone (CEF), have largely retained their effectiveness, with susceptibility ~70%, emphasizing these antibiotics as promising options for treatment (Table 5).

## 3. Discussion

AMR and the emergence of difficult-to-treat bacterial infections (i.e., infections caused by bacteria resistant to most first-line agents) has emerged as one of the critical public health issues of the 21st century [30]. While the spread of MDR bacteria is a global phenomenon—including both human and animal medicine sectors—low- and middle-income countries bear a disproportionate burden of the morbidity and mortality associated with AMR [17,31]. According to the recently published WHO GLASS report, in 2023, one in six laboratory-confirmed bacterial infections was resistant to antibiotics, with urinary tract infections (~one out of three infections) and bloodstream infections (~one out of six infections) being the most commonly affected [32]. Furthermore, the report also highlighted that—although the number of participating countries has increased four-fold in the last decade—regions with lower AMR surveillance coverage were associated with higher levels of AMR [32]. As a country of interest, Pakistan has particular characteristics which make it especially vulnerable to the detrimental effects of AMR [33,34]. On the one hand, due to widespread economic scarcity, the majority of the population is unable to afford the costs of a consultation with a general practitioner or other healthcare specialist [35]. In addition, due to the high rate of illiteracy (~30%)—which has both direct and indirect effects on population health literacy and agency—increasing awareness towards the prudent use of antimicrobials has proven to be a daunting task on a population-level scale [36]. Access to healthcare is also limited by the lack of credible healthcare professionals (1 physician per 1300 patients), and the structure of the healthcare services, which are vulnerable to service bottlenecks and breakdowns [19,37]. Furthermore, previous reports have indicated that there is a limited understanding among healthcare professionals related to the management of bacterial infections—and their personal roles in curbing AMR—in Pakistan [38].

As a result, there is a consistent creep in the management of bacterial infections by the population towards self-medication with antimicrobials (SMA; obtained without a prescription from pharmacies or other sources), or patients only receiving medical attention when their infections become severely debilitating or life-threatening [19,38]. In a recent cross-sectional study, the prevalence of SMA in Pakistan was reported at 32.5%, with coughs or colds, a runny nose, flu or sore throat, diarrhea, or fevers being cited as the main reasons for taking the drugs; furthermore, ciprofloxacin was the most commonly used drug for SMA. As SMA is one of the main hallmarks of the development of AMR—which was further compounded by the COVID-19 pandemic—the introduction of community-based AMS interventions would be of pivotal importance to promote the judicious use of these medicines [39,40]. In recent years, Pakistan has demonstrated a heightened commitment towards initiatives to curb AMR and its consequences, including the participation in WHO’s GLASS and the release of the country’s own National Action Plan (NAP) on AMR in 2017, with aspects covering human and animal medicine, agriculture, and environmental contexts [37,41]. The introduction of the WHO’s Access, Watch, and Reserve into practice has also given new perspectives on the judicious use of antibiotics [39]. Furthermore, substantial focus has been given to facilitating prudent IPC within the country’s healthcare providers [42].

A recent study by Jamil et al. aimed to identify the (institutional/systemic/professional) barriers to implementing effective AMS programs in Pakistan, from the perspective of healthcare professionals. Their results highlighted the insufficiency of trained human resources, a lack of hospital AMS policies, high patient load, clinicians resistant to changing habitual prescribing behaviors, and inadequate laboratory support as perceived barriers [43]. The latter underscores one of the major roadblocks towards optimizing the rational usage of antimicrobials, which is the availability of reliable and curated susceptibility data; as the country consists of regions with considerably different economic development levels and a diverse patient pool, this may require attention when deciding on empirical treatment options, especially if limited microbiology laboratory capacities exist [44]. Our current study aimed to assess the rates of bacterial AMR in three major healthcare facilities—with dissimilar profiles and patient populations—in Northern Punjab, Pakistan, to generate a comprehensive regional, data-driven antibiogram, stratified by patient and institutional characteristics, to support empirical treatment decisions for clinicians in a regional context. Our study utilized CLSI guidelines in susceptibility testing and reporting, which are validated and internationally recognized, based on the availability and the sample size of the bacterial isolates during the timeframe of the study. By linking microbiological trends to clinical decision-making, this study underscores the importance of dynamic stewardship strategies in a local context, to ensure timely, effective, and targeted antimicrobial therapy. In parallel, to further triangulate the available evidence, the nature of antimicrobial prescription practices in these settings were also assessed, which may directly contribute to worsening resistance rates, both regionally and nationally.

Our study highlighted the pathogenic role of *S. aureus* and *Streptococcus* spp. among Gram-positive bacteria and *E. coli*, *Klebsiella* spp., and *P. aeruginosa* among Gram-negative bacteria within the study period, which is consistent with previous studies from the region; however, the relevance of pathogens causing severe infections—often associated with inadequate sanitation and food/water hygiene—such as *Campylobacter* spp., *Shigella* spp., enteric *Salmonella* spp., and *V. cholerae*, was also underlined by our results. Nonetheless, the level and technological capacities of clinical microbiology laboratories crucially influence the catchment rates of rarely occurring bacterial species and the determination of their susceptibility profiles [45].

Taking susceptibility data and AB utilization data together highlights several stewardship-relevant themes in the studied healthcare settings. The most commonly prescribed AB in all age groups, healthcare settings, and departments was ceftriaxone (which is only available as an intravenous or intramuscular injection), a 3GC in the WHO “Watch” AB group with broad spectrum activity, commonly used in the empiric treatment of Gram-negative infections; the overuse of ceftriaxone has also been described elsewhere [46]. Furthermore, a recent study by Afzal et al. has emphasized that the overuse of ceftriaxone (vs. other more narrow-spectrum drugs) may be associated with faulty AB procurement policies in Pakistan, which favor its greater procurement/use against AWaRe “Access” group drugs; this paper suggests that “procurement-side” AMS considerations are also warranted for the judicious prescription of ABs [47]. Other commonly prescribed ABs were fluoroquinolones, meropenem, and metronidazole. The AB susceptibility of both Gram-negative and Gram-positive bacteria highlighted meropenem as the most effective agent across a broad range of pathogens, underscoring its critical role in treating resistant infections. For infections caused by Gram-negative bacteria, the preservation of carbapenems and colistin for proven MDR isolates is crucial to avoid accelerating the development of resistance [48]. However, due to the high rates of Enterobacterales with resistance to 3GCs and carriage of extended-spectrum beta-lactamase (ESBL) genes, the use of carbapenems (only available parenterally), out of necessity, has increased substantially, which has led to a considerable increase in carbapenem non-susceptible isolates globally [49]. Overall, the findings of this study are largely consistent with previously published data, i.e., the majority of ABs used were among the “Watch” and “Reserve” group drugs, although notable differences in certain susceptibility rates emphasize the influence of local prescribing patterns, IPC, and genetic resistance determinants. In the case of Gram-positive bacteria, susceptibility was largely retained towards aminoglycosides, vancomycin, and linezolid, which validates their role as empiric options where MRSA or other resistant Gram-positive cocci are a concern. In contrast, the poor performance of fluoroquinolones (available in both *per os* and parenteral formulations), especially ciprofloxacin in both Gram-positive (S%: 25–75%) and Gram-negative (S%: 35–70%) infections, cautions strongly against their routine empiric use in serious infections in these settings; many guidelines and recommendations also suggest limiting their use—and removing them from the list of first-line ABs—due to “black box warnings” and the risk of serious adverse events associated with their use [50]. Aminoglycosides retain some utility in combination regimens, although nephrotoxicity risks necessitate careful monitoring. On the other hand, metronidazole—available in both *per os* and parenteral formulations—is relevant in the treatment of infections caused by strict anaerobic bacteria, often seen after gynecological and abdominal surgeries [51]; the notable rate of metronidazole prescriptions in our context may also signal their unnecessary use. The low rate of using narrower-spectrum ABs, demonstrated in the three hospitals, may be attributed to—among other things—the preferences of the clinicians and/or the patients, the availability and procurement processes of the specific drugs, the limited awareness of prescribers towards sequential therapy, or strong adherence to habitual prescribing behaviors [52]; nonetheless, the usage rate of broad-spectrum ABs may be considered a relevant indicator of prescription quality, and “low-hanging fruit” for hospital-based AMS. The role and insights of hospital/clinical pharmacists may be invaluable to curb inappropriate AB prescriptions, and to optimize their use when its indeed necessary [53].

A point prevalence survey (PPS) among eleven Pakistani hospitals between 2020 and 2021—on account of the COVID-19 pandemic—has highlighted that almost two-thirds (64.6%) of hospitalized patients received antibiotics, administered empirically in most cases (97.9%), commonly associated with pneumonia, CNS infections, and gastro-intestinal tract diseases as indications. Similarly to our study, the overuse of ceftriaxone and metronidazole—and other broad-spectrum agents more generally—were highlighted, in addition to the prolonged used of surgical prophylaxis [54].

Our results provide valuable insights into the susceptibility of clinically relevant bacteria to commonly used antibiotics; the variability observed also highlights the necessity of continuous local surveillance, regular antibiogram updates, and the integration of the resulting data into empiric therapy guidelines. While several single-center or regional studies have described similar resistance trends, comprehensive multi-institutional data, based on reliable laboratory support, is scarce in the Pakistani context. Our findings indicate the retained high susceptibility to vancomycin and linezolid among *S. aureus* (including MRSA) isolates, which are similar to the findings from Iftikhar et al. [23], who also highlighted the efficacy of glycopeptides in the empirical treatment of Gram-positive infections in Peshawar, Pakistan. On the other hand, the increasing incidence of MRSA and MR-*S. epidermidis* was shown in the region by Handa et al. [55], largely mediated by the expression of the *mecA* gene; their study also underscored the necessity of cautious de-escalation in therapy away from “last-resort” drugs, once isolates are confirmed to be methicillin-susceptible. In our study, *S. pneumoniae* exhibited high susceptibility to ceftriaxone, vancomycin, and linezolid; these results were verified by a previous report by Tran-Quang et al. [56], where the rate of resistance to ceftriaxone was 16.9%. These findings reinforce the role of ceftriaxone and glycopeptides as frontline options for invasive pneumococcal disease, though the risk of rising ceftriaxone resistance, due to its excessive use and horizontal gene transfer, remains a concern, as reports from certain regions have noted an increase in resistance among *S. pneumoniae*, potentially due to excessive antibiotic use and horizontal gene transfer [57]. In contrast, *S. pyogenes* also showed remarkable sensitivity to most tested antibiotics, reinforcing the earlier findings of Lehtinen et al., that they remain effective treatment options [58].

When it comes to Gram-negative bacteria, *E. coli* has shown retained susceptibility to piperacillin/tazobactam, ceftriaxone, and carbapenems, whereas Harris et al. showed considerable non-susceptibility rates to 3GCs [13]; furthermore, the study of Hassan et al. showed imipenem and colistin as most effective antibiotics against *E. coli* infections [59]. The findings are supported by Fatima et al., where fosfomycin, colistin, and carbapenems retained their effectiveness against urinary *E. coli* and *Klebsiella* spp. isolates, while commonly used drugs in empiric treatment (i.e., fluoroquinolones, trimethoprim–sulfamethoxazole, 3GCs) had considerable non-susceptibility rates (>70%) in Karachi [60]. The study by Khan et al. in Lower Dir, Khyber Pakhtunkhwa, also verifies this. In their case, both urinary *E. coli* and *Klebsiella* spp. isolates showed ~100% susceptibility to imipenem, with almost uniform resistance against cefuroxime and ciprofloxacin, respectively [61]. Iqbal et al. reported an even bleaker picture, corresponding to pediatric urinary tract infections from two hospitals in Lahore: in the context of *E. coli* and *Klebsiella* spp., colistin retained effectiveness (100%), while meropenem and fosfomycin had susceptibility rates ~70%; on the other hand, most 3GCs and beta-lactam and beta-lactamase–inhibitor combinations showed non-susceptibility ≥90% [62]. Khan et al. reported on the susceptibility of pus aspirate isolates in chronic suppurative otitis media from a tertiary care hospital in Rawalpindi; their study highlighted the lower susceptibility of *P. aeruginosa* towards beta-lactams (such as piperacillin/tazobactam) and the limited effectiveness of macrolides and fluoroquinolones against *S. aureus*, which was also demonstrated in our setting [63].

Although our findings align with earlier reports regarding the predominance of ceftriaxone use and rising rates of resistance in common pathogens in human infections, our study adds value by contextualizing these patterns across multiple institutions, which have different clinical/therapeutic profiles and serve substantially different patient populations, highlighting the health equity aspects of AMR. This study thus serves as a preliminary validation of prior localized evidence on a broader scale, emphasizing the systemic nature of the concern of AMR and underscoring the urgent need for coordinated interventions, both in a regional (i.e., Punjab) and national context. Regarding this study’s multicenter design, isolates originating from a rich variety of sources and the use of standardized CLSI guidelines during susceptibility testing are notable strengths that allow us to provide valuable insights into the patterns of antimicrobial use and resistance with a regional focus. Several limitations must be acknowledged, which may considerably influence the external validity of our findings. Our six-month, cross-sectional study has provided valuable insight into the patterns of antimicrobial use and resistance; however, it is unsuitable to assess the temporal dynamics of these resistance trends, which is a major limitation from the standpoint of surveillance. To this end, longitudinal surveillance would be crucial to assess changes over time, spot emerging resistance hotspots, and evaluate the impact of AMS, if present. Effective susceptibility reporting—per CLSI—necessitates *n* ≥ 30 isolates per species, which was not always available in our case; these instances were noted in our results to highlight their potential unreliability. Furthermore, molecular epidemiological analyses—such as the genetic characterization of extended-spectrum β-lactamase (ESBL) and carbapenemase production in Gram-negative bacteria—would be essential to identify the key resistance mechanisms and potential clonal spread within and across institutions. While the preliminary nature of our findings must be acknowledged, they nonetheless provide useful insights into antimicrobial resistance patterns in Punjab, contributing to the development of regional antibiograms, and highlight trends that warrant further investigation. These findings underscore the urgent need for tailored antibiotic stewardship programs and continuous surveillance to optimize antibiotic use and effectively combat resistance.

## 4. Materials and Methods

### 4.1. Study Design, Setting, and Duration

This retrospective, cross-sectional, multicenter study was conducted to investigate antimicrobial susceptibility patterns and related patient data across three tertiary care hospitals (Hospitals 1–3) in Northern Punjab, Pakistan. Hospital 1 was a large public sector tertiary care facility, with an over 1000 bed capacity, offering a broad range of medical and surgical specialties, including cardiology, nephrology, neurosurgery, oncology, and trauma care, supported by modern diagnostic and laboratory services. Similarly, Hospital 2 was a major public tertiary hospital, with approximately 1500 beds, providing comprehensive healthcare services across internal medicine, general surgery, orthopedics, pediatrics, gynecology and obstetrics, and critical care. Both Hospital 1 and Hospital 2 primarily serve low- to middle-income populations from Lahore and surrounding districts, while also receiving referrals from underserved rural regions of Northern and Central Punjab, where specialized healthcare facilities are limited. The University of Lahore Teaching Hospital (Hospital 3) is a modern private tertiary care teaching hospital, with a bed capacity of ~500 beds, affiliated with a medical and dental faculty. It offers advanced services in specialties such as cardiology, neurology, gastroenterology, oncology, and minimally invasive surgery, supported by well-equipped intensive care units and diagnostic facilities. Unlike the public hospitals, Hospital 3 mainly caters to middle- and high-income patients, who seek improved service quality, shorter waiting times, and access to advanced medical technologies, though it also attracts complex referral cases from across the Punjab region. The study was conducted over a six-month period, spanning from February to August 2024, enabling the collection and analysis of data across different seasonal patterns and patient demographics.

### 4.2. Data Collection and Sample Size Determination

Data were collected from electronic health records (EHRs) and laboratory information system data using a pre-designed checklist. The analysis focused on bacterial isolates obtained from patients with suspected bacterial infections, including inpatients and outpatients treated at one of the participating healthcare facilities. In addition to the institutional affiliation and microbiology laboratory findings (including specimen type, isolates, and susceptibility results), data on socio-demographic information (age and sex), departments where they received treatment, outpatient pharmacy prescriptions (focusing on antibiotics, evaluated based on the WHO “AWaRe” classification), and inpatient charts were also collected. Data received and used by the researchers were de-identified, per de-identification standards; therefore, information could not be traced back to patients/individuals treated at the healthcare facility.

### 4.3. Inclusion and Exclusion Criteria and Sample Size Determination

This study included data from patients of all ages presenting with suspected or confirmed bacterial infections during the designated study period. Only the first isolate per patient was included in the study; however, isolates with different antibiotic susceptibility patterns were considered as different individual isolates [63]. Patients with polymicrobial (mixed) culture results were excluded from the analysis of susceptibility testing results [61]. Furthermore, microbiological and patient data were excluded if the isolate was deemed not clinically significant (normal flora or contaminants) or if the patient presented with chronic bacterial (e.g., tuberculosis) or viral (e.g., HIV/AIDS) infections, a severe immunosuppressive state, or other severe conditions (e.g., end-stage renal disease). Moreover, if any of the patients made any prior declaration of his/her non-consent for participation in any type of study or research, the data file was excluded. The sample size of this study was determined according to the Clinical and Laboratory Standards Institute (CLSI) guidelines [64].

### 4.4. Sample Collection and Processing

Clinical specimens were collected using standard aseptic techniques to maintain the integrity of the samples. Blood cultures were obtained from two separate sites after skin preparation with 70% alcohol (Sigma-Aldrich, St. Louis, MO, USA) and iodine/chlorhexidine (Sigma-Aldrich, St. Louis, MO, USA), collecting 10–20 mL (adults) or 1–5 mL (pediatrics) per bottle (BOENMED, Suzhou, China). Midstream urine specimens were collected after cleansing the urethral area, while catheter samples were taken from the sampling port. Sputum, stool, wound/pus, cerebrospinal fluid specimens, throat, and genital samples were collected using appropriate sterile methods specific to each type, avoiding contamination. All samples were promptly transported to the laboratory for microbiological analysis, and processing of samples was carried out within 24 h of receipt by the laboratory. Samples were inoculated onto appropriate culture media, such as blood agar, MacConkey agar, or chocolate agar, depending on the suspected organism and/or relevant sample type. Inoculated plates were incubated at 35–37 °C for 18–24 h under aerobic or anaerobic conditions, as required. After incubation, colony morphology, hemolytic patterns, and pigmentation were visually observed and examined under a colony microscope. Preliminary identification was performed using Gram staining. Further confirmation was performed through biochemical tests (e.g., catalase, coagulase, oxidase, API strips), automated identification systems (VITEK, MALDI-TOF), or molecular methods, where applicable [65].

### 4.5. Antimicrobial Susceptibility Testing and Reporting of Results

Antimicrobial susceptibility testing was performed following CLSI guidelines, valid at the time of the interpretation [66]; the procedure included the preparation of pure bacterial cultures on selective media and the standardization of bacterial suspensions to specific turbidity before testing. Depending on the standardized method for the bacterial species in question, the Kirby–Bauer disk diffusion method, broth microdilution, or agar dilution techniques were utilized. Plates were incubated under appropriate conditions depending on the bacterial species—typically at 35 ± 2 °C for 16–18 h for non-fastidious organisms, and up to 24 h for fastidious species—using relevant atmospheric requirements (aerobic, 5% CO_2_, or anaerobic as indicated). Mueller–Hinton agar (Oxoid, Basingstoke, UK) was used for disk diffusion testing, supplemented with 5% defibrinated sheep blood or chocolate agar (Oxoid, Basingstoke, UK) as required for fastidious bacteria [63]. Antibiotic disks (Oxoid, Basingstoke, UK) were applied aseptically, and inhibition zones were measured in millimeters using a calibrated ruler. Minimum inhibitory concentrations (MICs) were determined using the broth microdilution method, using cation-adjusted Mueller–Hinton broth (Oxoid, Basingstoke, UK) in sterile microtiter plates (Sigma-Aldrich, St. Louis, MO, USA). Results were interpreted according to CLSI breakpoints (latest edition at the time of the study) to classify isolates as susceptible (S) or resistant (R) to each antibiotic tested [66]. During interpretation, intrinsic resistance and expected resistant phenotypes of relevant bacterial species were taken into account [67]. Methicillin resistance was detected using mannitol salt agar and cefoxitin disks [68].

During analyses, quality control (QC) strains were used to validate the accuracy of the testing methods, as follows: *S. aureus* ATCC 29213, *S. pyogenes* ATCC 12384, *E. faecalis* ATCC 29212, *P. mirabilis* ATCC 35659, *E. coli* ATCC 25922, *K. pneumoniae* ATCC 49619, *P. aeruginosa* ATCC 27853, and *A. baumannii* ATCC 19606 [69].

### 4.6. Data Management and Statistical Analysis

Data collected during the study were initially handled in a Microsoft Excel 2013 (Microsoft Corp., Redmond, WA, USA) spreadsheet, which was then transferred to the Statistical Package for the Social Sciences (SPSS) v.22.0 (IBM Corp., Endicott, NY, USA) for analysis. During descriptive statistics, all categorical variables were described in terms of frequencies and percentages (*n*, %).

### 4.7. Ethical Considerations

This study was conducted in accordance with the Declaration of Helsinki (1975, last revised in 2013) and relevant national and institutional ethical standards. Prior to data collection, ethical approval was obtained from the Board of Advanced Studies and Research at the University of Lahore (approval number IREC-2024-0211; date: 29 January 2024) and the Institutional Review Boards (IRBs) of the three participating hospitals: Hospital 1 (approval number ERB162/1/04-04-2024/S1ERB; date: 4 April 2024), Hospital 2 (approval number 02-TERC/NHRC-SZH/EXT/SC/440; date: 22 February 2024), and Hospital 3 (approval number ERC/04/24/02; date: 22 February 2024). The data reviewed was a secondary analysis of existing data, which did not involve intervention or interaction with human subjects. Furthermore, data received and used by the researchers were de-identified, per de-identification standards; therefore information could not be traced back to patients/individuals treated at the healthcare facility. This study followed established ethical guidelines for data handling, storage, and reporting to ensure patient confidentiality.

## Figures and Tables

**Table 1:** Socio-demographic characteristics of the patients affected by bacterial infections during the study period.

Variable	*n* (%)
**Gender**	
Male	285 (58.8%)
Female	200 (41.2%)
**Age group**	
<20 years	80 (16.5%)
20–39 years	150 (30.9%)
40–60 years	170 (35.1%)
>60 years	85 (17.5%)
**Healthcare Facility**	
Hospital 1 (Public)	120 (24.7%)
Hospital 2 (Public)	130 (26.8%)
Hospital 3 (Private)	235 (48.5%)
**Patient status during treatment**	
Inpatient	310 (63.9%)
Outpatient	175 (36.1%)
**Hospital department associated with treatment**	
Cardiology	50 (10.3%)
Gastroenterology	40 (8.2%)
Gynecology	55 (11.3%)
Kidney and Pancreas	35 (7.2%)
Internal medicine	60 (12.4%)
Oncology	45 (9.3%)
Orthopedics	30 (6.2%)
Pediatrics	50 (10.3%)
Pulmonology	40 (8.2%)
Surgical	50 (10.3%)
Urology	30 (6.2%)

**Table 2:** Frequency and distribution of bacterial isolates from clinically relevant infections.

Bacterial Groups	Bacterial Genera/Species	*n* (%)
**Gram-positive bacteria**		**291 (60.00%)**
	*Staphylococcus aureus*	59 (12.16%)
	*Streptococcus pneumoniae*	50 (10.31%)
	*Streptococcus pyogenes*	35 (7.22%)
	*Enterococcus faecalis*	28 (5.77%)
	*Bacillus subtilis*	27 (5.57%)
	*Bacillus cereus*	24 (4.95%)
	*Clostridium perfringens*	22 (4.54%)
	*Listeria monocytogenes*	20 (4.12%)
	*Corynebacterium diphtheriae*	15 (3.09%)
	*Actinomyces* spp.	11 (2.27%)
**Gram-negative bacteria**		**194 (40.00%)**
	*Escherichia coli*	68 (14.02%)
	*Pseudomonas aeruginosa*	28 (5.77%)
	*Klebsiella pneumoniae*	18 (3.71%)
	*Salmonella Enterica*	17 (3.51%)
	*Haemophilus influenzae*	13 (2.68%)
	*Neisseria gonorrhoeae*	8 (1.65%)
	*Neisseria meningitidis*	8 (1.65%)
	*Vibrio cholerae*	8 (1.65%)
	*Proteus mirabilis*	8 (1.65%)
	*Campylobacter jejuni*	7 (1.44%)
	*Acinetobacter baumannii*	7 (1.44%)
	*Shigella* spp.	4 (0.82%)

Bacterial species with *n* ≥ 30 are denoted in green, 30 > *n* > 10 are denoted in yellow, while *n* ≤ 10 are denoted in red.

**Table 3:** Frequency and characteristics of antibiotic use in different settings, based on the number of antimicrobial prescriptions.

	Ranks Based on Frequency of Antibiotics Prescribed				
	**1.**	2.	3.	**4.**	**5.**
Based on department					
**Cardiology** (*n* = 75)	CRO (*n* = 24; 32.0%)	CIP (*n* = 16; 21.3%)	PIP/TAZO (*n* = 14; 18.7%)	MER (*n* = 12; 16.0%)	VAN (*n* = 9; 12.0%)
**Gastroenterology** (*n* = 237)	MET (*n* = 90; 38.0%)	CRO (*n* = 75; 31.6%)	CIP (*n* = 30; 12.7%)	PIP/TAZO (*n* = 24; 10.1%)	MOX (*n* = 18; 7.6%)
**Gynecology** (*n* = 88)	CRO (*n* = 39; 44.3%)	MET (*n* = 23; 26.1%)	CIP (*n* = 11; 12.5%)	PIP/TAZO (*n* = 9; 10.2%)	CAZ (*n* = 6; 6.8%)
**Kidney and Pancreas** (*n* = 55)	MER (*n* = 18; 32.7%)	CRO (*n* = 12; 21.8%)	PIP/TAZO (*n* = 11; 20.0%)	AMK (*n* = 8; 14.5%)	VAN (*n* = 6; 10.9%)
**Internal Medicine** (*n* = 156)	CRO (*n* = 72; 46.2%)	MET (*n* = 27; 17.3%)	CIP (*n* = 23; 14.7%)	MOX (*n* = 18; 11.5%)	PIP/TAZO (*n* = 16; 10.3%)
**Oncology** (*n* = 174)	CRO (*n* = 27; 36.5%)	MER (*n* = 18; 24.3%)	PIP/TAZO (*n* = 11; 14.9%)	VAN (*n* = 9; 12.2%)	AMK (*n* = 9; 12.2%)
**Orthopedics** (*n* = 58)	CRO (*n* = 18; 31.0%)	CIP (*n* = 13; 22.4%)	AMK (*n* = 12; 20.7%)	PIP/TAZO (*n* = 9; 15.5%)	CAZ (*n* = 6; 10.3%)
**Pediatrics** (*n* = 129)	CRO (*n* = 18; 31.0%)	CTX (*n* = 27; 20.9%)	AMK (*n* = 18; 14.0%)	MET (*n* = 15; 11.6%)	CIP (*n* = 9; 7.0%)
**Pulmonology** (*n* = 84)	CRO (*n* = 33; 39.3%)	CIP (*n* = 23; 27.4%)	MOX (*n* = 13; 15.5%)	MER (*n* = 9; 10.7%)	PIP/TAZO (*n* = 6; 7.1%)
**Surgery** (*n* = 128)	CRO (*n* = 47; 36.7%)	MET (*n* = 30; 23.4%)	PIP/TAZO (*n* = 23; 18.0%)	CIP (*n* = 16; 12.5%)	CAZ (*n* = 12; 9.4%)
**Urology** (*n* = 64)	CIP (*n* = 26; 40.6%)	CRO (*n* = 15; 23.4%)	PIP/TAZO (*n* = 9; 14.1%)	AMK (*n* = 8; 12.5%)	MOX (*n* = 6; 9.4%)
**Based on age groups**					
**<20 years** (*n* = 167)	CRO (*n* = 84; 50.3%)	MET (*n* = 22; 13,2%)	CIP (*n* = 17; 10.2%)	MOX (*n* = 11; 6.6%)	AMK (*n* = 10; 6.0%)
**20–39 years** (*n* = 373)	CRO (*n* = 139; 37.3%)	MET (*n* = 70; 18.8%)	CIP (*n* = 67; 18.0%)	PIP/TAZO (*n* = 33; 8.8%)	MOX (*n* = 19; 5.1%)
**40–60 years** (*n* = 364)	CRO (*n* = 127; 34.9%)	MET (*n* = 65; 17.9%)	CIP (*n* = 59; 16.2%)	PIP/TAZO (*n* = 40; 11.0%)	MOX (*n* = 20; 5.5%)
**>60 years** (*n* = 244)	CRO (*n* = 72; 29.5%)	PIP/TAZO (*n* = 46; 18.9%)	MET (*n* = 28; 11.5%)	MER (*n* = 26; 10.7%)	CIP (*n* = 24; 9.8%)
**Type of hospital**					
Public (*n* = 689)	CRO (*n* = 255; 37.0%)	MET (*n* = 118; 17.1%)	CIP (*n* = 95; 13.8%)	PIP/TAZO (*n* = 72; 10.4%)	MOX (*n* = 32; 4.6%)
Private (*n* = 459)	CRO (*n* = 167; 36.4%)	CIP (*n* = 72; 15.7%)	MET (*n* = 67; 14.6%)	AMK (*n* = 35; 7.6%)	PIP/TAZO (*n* = 60; 13.1%)

Abbreviations: AMK: amikacin; CRO: ceftriaxone; CIP: ciprofloxacin; CTX: cefotaxime; MER: meropenem; MET: metronidazole; MOX: moxifloxacin; PIP/TAZO: piperacillin/tazobactam; VAN: vancomycin; CAZ: ceftazidime. Color scheme: green: WHO “Access” antibiotics; yellow: WHO “Watch” antibiotic; red: WHO “Reserve” antibiotics. Only the first five ranks are shown for each ward.

**Table 4:** Ratio of susceptible isolates (S%) among clinically relevant Gram-positive bacteria isolated in the study.

Species	CRO (%)	CEF (%)	CXM (%)	CAZ (%)	MER (%)	AZI (%)	AMK (%)	LZD (%)	CIP (%)	MOX (%)	VAN (%)
*S. aureus*	75	75	75	75	75	70	80	95	65	75	95
*S. pyogenes*	90	92	90	82	95	88	85	94	60	70	100
*S. pneumoniae*	85	88	85	78	91	80	75	92	70	80	90
*E. faecalis*	60	62	58	52	87	55	50	68	40	30	70
*B. cereus*	55	50	48	45	80	40	45	32	50	40	30
*B. subtilis*	40	38	35	32	91	30	35	30	30	25	20
*C. diphtheriae*	65	58	63	65	89	60	60	58	55	50	55
*L. monocytogenes*	80	78	72	74	90	77	70	82	75	65	85
*C. perfringens*	50	45	42	40	95	35	n.r.	20	30	25	20
*Actinomyces* spp.	45	42	38	37	85	33	n.r.	40	35	30	35

Abbreviations: AMK: amikacin; AZI: azithromycin; CRO: ceftriaxone; CIP: ciprofloxacin; CTX: cefotaxime; CXM: cefuroxime; LZD: linezolid; MER: meropenem; MET: metronidazole; MOX: moxifloxacin; VAN: vancomycin; CAZ: ceftazidime; n.r.: not relevant. Bacterial species with *n* ≥ 30 are denoted in green, 30 > *n* > 10 are denoted in yellow.

**Table 5:** Ratio of susceptible isolates (S%) among clinically relevant Gram-negative bacteria isolated in the study.

Species	CRO (%)	CEF (%)	CXM (%)	CAZ (%)	PIP/TAZO (%)	MER (%)	AZI (%)	AMK (%)	CIP (%)	MOX (%)
*E. coli*	70	65	70	75	90	90	n.r.	80	60	75
*K. pneumoniae*	60	55	65	65	70	90	n.r.	55	45	50
*P. mirabilis*	70	60	70	75	80	89	n.r.	65	50	60
*S. Enterica*	65	60	65	70	80	91	55	60	55	50
*Shigella* spp.	85	80	85	80	90	85	75	75	65	70
*C. jejuni*	80	40	50	55	30	91	45	n.r.	40	35
*V. cholerae*	60	65	60	65	70	95	55	55	50	40
*A. baumannii*	n.r.	n.r.	n.r.	n.r.	n.r.	92	n.r.	40	35	30
*P. aeruginosa*	n.r.	n.r.	n.r.	60	45	95	n.r.	50	65	60
*H. influenzae*	75	80	75	85	85	85	n.r.	n.r.	65	60
*N. meningitidis*	80	75	80	70	85	80	75	75	70	65
*N. gonorrhoeae*	60	70	80	65	75	87	70	70	65	55

Abbreviations: AMK: amikacin; AZI: azithromycin; CEF: cefoperazone; CRO: ceftriaxone; CIP: ciprofloxacin; CTX: cefotaxime; CXM: cefuroxime; LZD: linezolid; MER: meropenem; MET: metronidazole; MOX: moxifloxacin; PIP/TAZO: piperacillin/tazobactam; VAN: vancomycin; CAZ: ceftazidime; n.r.: not relevant. Bacterial species with *n* ≥ 30 are denoted in green, 30 > *n* > 10 are denoted in yellow, while *n* ≤ 10 are denoted in red.

## Data Availability

All data generated during the study are presented in this paper.

## Supplementary Materials

The following supporting information can be downloaded at: https://www.mdpi.com/article/10.3390/antibiotics14111154/s1, Figure S1. Distribution of bacterial isolates from clinically relevant infections, according to specimen type of origin.
