# Supplementary material for: Multicentric Surveillance of Antimicrobial Resistance to Generate Data-Driven Regional Antibiograms: A Laboratory-Based Cross-Sectional Study in Pakistan
Source: Antibiotics (Basel). 2025 Nov 14;14(11):1154. doi: 10.3390/antibiotics14111154 (PMC12649520; doi:10.3390/antibiotics14111154)
Supplement: Supplementary file 1 [file antibiotics-14-01154-s001.zip › antibiotics-3875093-supplementary.pdf]

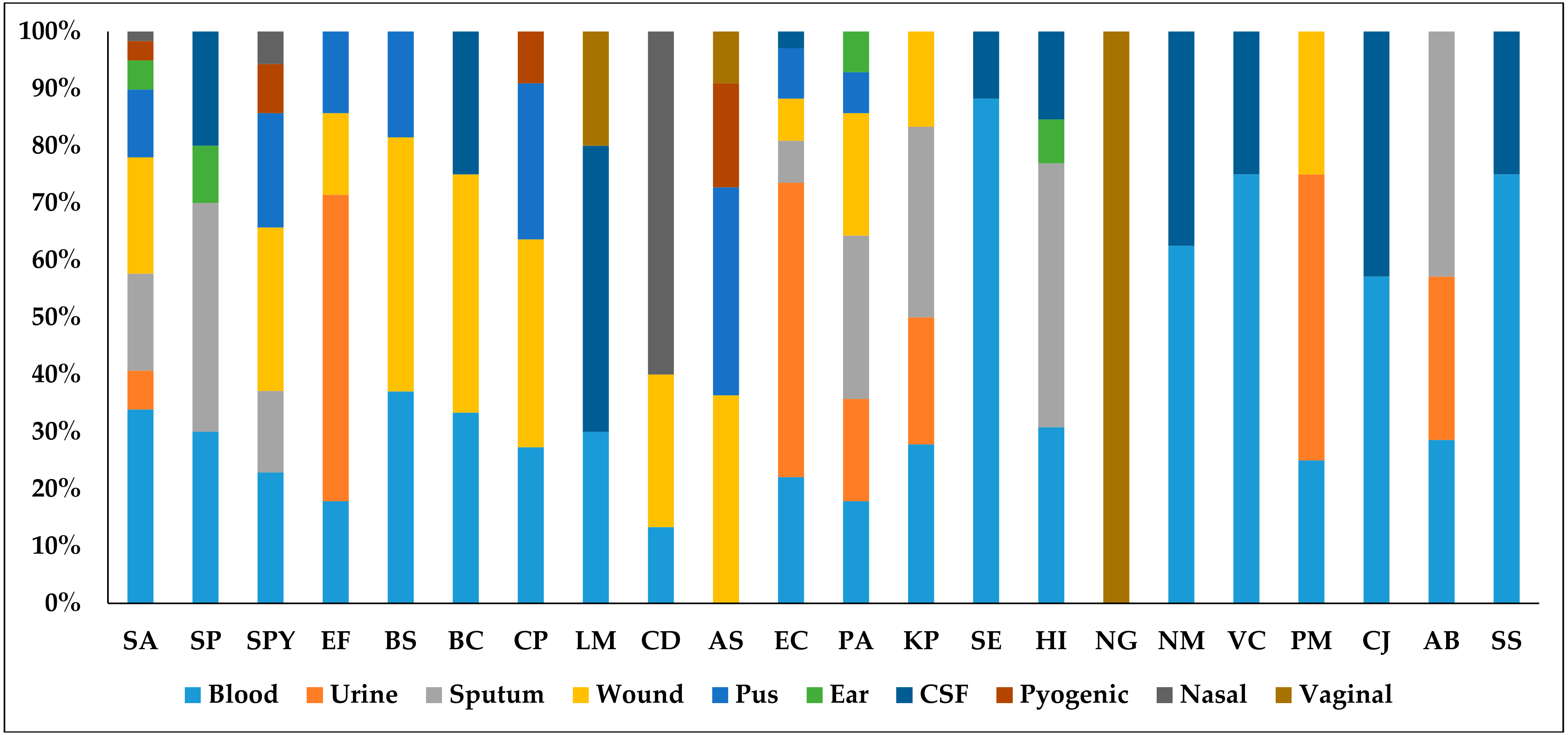

Figure S1. Distribution of bacterial isolates from clinically relevant infections, according to specimen type of origin.

Abbreviations: SA: *Staphylococcus aureus*; SP: *Streptococcus pneumoniae*; SPY: *Streptococcus pyogenes*; EF: *Enterococcus faecalis*; BS: *Bacillus subtilis*; BC: *Bacillus cereus*; CP: *Clostridium perfringens*; LM: *Listeria monocytogenes*; CD: *Corynebacterium diphtheriae*; AS: *Actinomyces spp.*; EC: *Escherichia coli*; PA: *Pseudomonas aeruginosa*; KP: *Klebsiella pneumoniae*; SE:*Salmonella enterica*; HI: *Haemophilus influenzae*; NG: *Neisseria gonorrhoeae*; NM: *Neisseria meningitidis*; VC: *Vibrio cholerae*; PM: *Proteus mirabilis*; CJ: *Campylobacter jejuni*; AB: *Acinetobacter baumannii*; SS: *Shigella spp.*.
